# Prevalence of SOS-mediated control of integron integrase expression as an adaptive trait of chromosomal and mobile integrons

**DOI:** 10.1186/1759-8753-2-6

**Published:** 2011-04-30

**Authors:** Guillaume Cambray, Neus Sanchez-Alberola, Susana Campoy, Émilie Guerin, Sandra Da Re, Bruno González-Zorn, Marie-Cécile Ploy, Jordi Barbé, Didier Mazel, Ivan Erill

**Affiliations:** 1 Institut Pasteur, Unité Plasticité du Génome Bactérien, CNRS URA 2171, 75015 Paris, France; 2 Departament de Genètica i de Microbiologia, Universitat Autònoma de Barcelona, 08193 Bellaterra, Spain; 3 Department of Biological Sciences, University of Maryland Baltimore County, Baltimore 21228, USA; 4 Université de Limoges, Faculté de Médecine, EA3175, INSERM, Equipe Avenir, Limoges 87000, France; 5 Departamento de Sanidad Animal, Facultad de Veterinaria, and VISAVET, Universidad Complutense de Madrid, 28040 Madrid, Spain

## Abstract

**Background:**

Integrons are found in hundreds of environmental bacterial species, but are mainly known as the agents responsible for the capture and spread of antibiotic-resistance determinants between Gram-negative pathogens. The SOS response is a regulatory network under control of the repressor protein LexA targeted at addressing DNA damage, thus promoting genetic variation in times of stress. We recently reported a direct link between the SOS response and the expression of integron integrases in *Vibrio cholerae *and a plasmid-borne class 1 mobile integron. SOS regulation enhances cassette swapping and capture in stressful conditions, while freezing the integron in steady environments. We conducted a systematic study of available integron integrase promoter sequences to analyze the extent of this relationship across the Bacteria domain.

**Results:**

Our results showed that LexA controls the expression of a large fraction of integron integrases by binding to *Escherichia coli*-like LexA binding sites. In addition, the results provide experimental validation of LexA control of the integrase gene for another *Vibrio *chromosomal integron and for a multiresistance plasmid harboring two integrons. There was a significant correlation between lack of LexA control and predicted inactivation of integrase genes, even though experimental evidence also indicates that LexA regulation may be lost to enhance expression of integron cassettes.

**Conclusions:**

Ancestral-state reconstruction on an integron integrase phylogeny led us to conclude that the ancestral integron was already regulated by LexA. The data also indicated that SOS regulation has been actively preserved in mobile integrons and large chromosomal integrons, suggesting that unregulated integrase activity is selected against. Nonetheless, additional adaptations have probably arisen to cope with unregulated integrase activity. Identifying them may be fundamental in deciphering the uneven distribution of integrons in the Bacteria domain.

## Background

**Figure 1:**
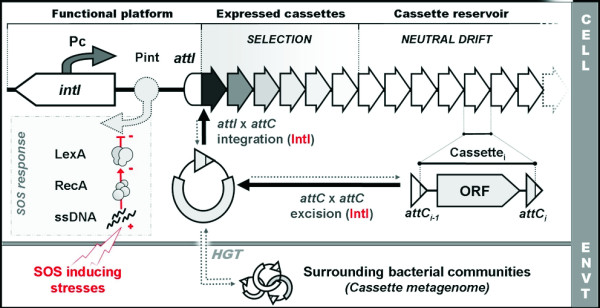
**Schematic organization of integrons**. The functional platform of integrons is constituted by an *intI *gene encoding an integrase, its own promoter *P_int_*, a cassette promoter *P_C_*, and a primary recombination site *attI*. The system maintains an array that can consist of more than 200 cassettes. Only the few first cassettes are strongly expressed by the *P_C _*promoter, as indicated by the fading fill color. Cassettes generally contain a promoterless open reading frame (ORF) flanked by two recombination sites termed *attC*. Cassettes can be excised from any position in the array through attC × attC recombination mediated by the integrase. The resulting circular intermediate can then be integrated by the integrase, preferentially at *attI*. Exogenous circular intermediates can also be integrated, owing to the low specificity of the integrase activity, rendering the system prone to horizontal transfer. The SOS response directly controls the expression of many integron integrases by binding of its repressor protein, LexA, to a target site in the *P_int _*promoter.

Integrons are bacterial genetic elements capable of incorporating exogenous and promoterless open reading frames (ORF), referred to as gene cassettes, by site-specific recombination (Figure 1). First described in the late 1980s in connection with the emergence of antibiotic resistance [1], integrons always contain three functional components: an integrase gene (*intI*), which mediates recombination; a primary recombination site (*attI*); and an outward-orientated promoter (*P_C_*) [2]. Cassette integrations occur mainly at the *attI *site, ensuring the correct expression of newly captured cassettes by placing them under the control of the *P_C _*promoter [3,4]. To date, two main subsets of integrons have been described. On the one hand, mobile integrons, also referred to as multiresistance integrons, contain relatively few (two to eight) cassettes, and collectively encode resistance to a broad spectrum of antibiotics [5-7]. They have been conventionally divided into five different classes according to their *intI *gene sequence: class 1 for *intI1*, class 2 for *intI2*, class 3 for *intI3*, class 4 for *intISXT *(formerly *intI9*) and class 5 for *intIHS *[8,9]. Mobile integrons are typically associated with transposons and conjugative plasmids, ensuring their dissemination across bacterial species. They are present mostly in the Proteobacteria, but have also been reported in other bacterial phyla, such as Gram-positive bacteria [9]. By contrast, chromosomal integrons have been identified in the genomes of many bacterial species [10]. Because their phylogeny reflects a predominant pattern of vertical inheritance, these integrons are not catalogued based on the class nomenclature described above, but according to their host species [8,9]. A subfamily of these, termed superintegrons (SIs), has been specifically identified in the *Vibrionaceae *and, to some extent, in the *Xanthomonadaceae *and *Pseudomonadaceae *[11-16]. Superintegrons typically encompass between 20 and 200 cassettes with species-specific sequence signatures [9], and seem to be ancient residents of the host genome [13]. Most of the genes in the superintegron cassettes are of unknown function [10], but some of them are related to existing resistance cassettes [17-20]. Although stable under laboratory conditions, superintegrons have been reported to be the most variable loci of *V. cholerae *natural isolates [12,21], suggesting that integron reorganization might be occasionally upregulated in natural environments. Integron integrases mediate recombination by interacting with single-stranded (ss) *attC *sites present in all reported cassettes, employing a unique, site-specific recombination process [22-24]. Despite the importance of integrons in the acquisition and spread of antibiotic-resistance determinants and, from a broader perspective, in bacterial adaptation, little was known about the regulatory control and dynamics of cassette recombination until recently, when we reported that the expression of the integron integrases in the *V. cholerae *superintegron and in a class 1 mobile integron was controlled by the SOS response [25].

The SOS response is a global regulatory network governed by a repressor protein (LexA) and principally targeted at addressing DNA damage [26,27]. LexA represses SOS genes by binding to highly specific binding sites present in their promoter regions. In *E. coli *and most β- and γ-Proteobacteria, these sites consist of a palindromic motif (CTGTatatatatACAG) 16 bp long, commonly known as the LexA box [26]. The SOS response is typically induced by the presence of ssDNA fragments, which can arise from a number of environmental stresses [28], but are typically linked to replication-fork stall caused by DNA lesions. These ssDNA fragments bind non-specifically to the universal recombination protein RecA [29], enabling it to promote LexA inactivation by autocatalytic cleavage [30], and thus inducing the SOS response. Up to 40 genes have been shown to be directly regulated by LexA in *E. coli *[31], encoding proteins that stabilize the replication fork, repair DNA, promote translesion synthesis and arrest cell division. Since its initial description in *E. coli *[26], the SOS response has been characterized in many other bacterial classes and phyla, and LexA has been shown to recognize markedly divergent motifs in different bacterial groups [27].

In recent years, the SOS response has been linked to clinically relevant phenotypes, such as the activation and dissemination of virulence factors carried in bacteriophages [32-34], transposons [35] pathogenicity islands [36] and in integrating conjugative elements encoding antibiotic-resistance genes [27,37,38]. Moreover, it has recently become established that some widely used antibiotics, such as fluoroquinolones, trimethoprim and β-lactams, are able to trigger SOS induction and are thus able to promote the dissemination of antibiotic-resistance genes [27,37,39-42]. This puts forward a positive feedback loop that has been suggested to have important consequences for the emergence and dissemination of antibiotic resistance [43]. Our recent work, showing a link between the SOS response and integrase-mediated recombination [25] further reinforces this line of reasoning. Such a link provides bacteria with an antibiotic-induced mechanism for gene acquisition, reorganization and dispersal. In hindsight, the coupling of genetic elements capable of cassette integration with a global response to stress comes out as an elegant and powerful pairing. Integrons can be seen as stockpiling agents of genetic diversity that, in addition, can tap into a huge and variable pool of cassettes through horizontal gene transfer from the surrounding bacterial communities (Figure 1) [10,44]. Nonetheless, efficient expression of these acquired traits is strongly dependent on integrase-mediated recombination. Newly acquired cassettes sitting in the proximal region of the integron are strongly expressed by the *P_C _*promoter, but they can be displaced gradually to distal parts of the integron by the incorporation of new cassettes, and can thus become partially silenced. Infrequent excision and integration events can also relocate cassettes, moving them to distal or proximal parts of the integron, and thus have the ability to reinstate previously acquired cassettes (Figure 1). The timing of all these events is therefore of fundamental importance, and depends on the regulatory systems controlling the expression of the integron integrase gene. In this context, the discovery of a link between the SOS response and integrase expression is an important first step towards unraveling the precise mechanisms controlling integrase expression.

In this study, we expanded on this recent connection between the SOS response and integron integrase expression by means of a systematic study of integron integrase promoter regions. By combining *in silico *and *in vitro *methods, we show that LexA control of integrase expression is a widespread phenomenon that arose very early in the evolutionary history of integrons and has since been maintained through positive selection in mobile integrons and large chromosomal integrons. We report a significant correlation between the loss of LexA control and integrase inactivation, indicating that unregulated recombination may be deleterious in these genetic elements. Exceptions to this rule suggest that secondary adaptations to tolerate unregulated integrase expression may have arisen in some clades, and that the identification of such adaptations might shed light onto the uneven distribution of integrons in the Bacteria domain. We discuss these findings for the adaptive dynamics of integrons, and their implications for the acquisition and dissemination of antibiotic-resistance determinants.

## Results and discussion

### Identification of LexA binding sites in *intI *promoters

**Figure 2:**
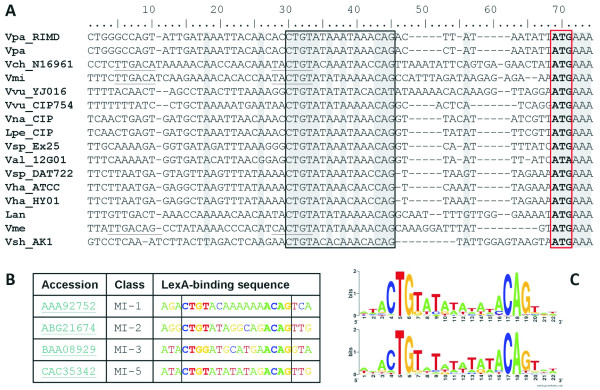
***In silico *analysis of integrase promoters**. **(A) **Alignment of representative promoter regions of Vibrionaceae *intIA *homologs. Putative LexA binding sequences are boxed, whereas putative σ70 promoter elements (-35 and -10) are underlined, and the translation start site of *intIA *is boxed and in bold type. The multiple sequence alignment was performed using CLUSTALW with default parameters [89]. **(B) **Representative examples of LexA binding sites identified upstream of different integrase genes from mobile integrons, with (1-5) denoting the integrase class. The provided accessors correspond to IntI proteins from: *Escherichia coli *pSa (AAA92752), *Providencia stuartii *ABR23a (ABG21674), *Serratia marcescens *AK9373 (BAA08929), *Vibrio cholerae *569B (AAC38424) and *Vibrio salmonicida *VS224 pRVS1 (CAC35342). **(C) **Sequence logos [100] of the profile used to search for β/**γ**-Proteobacteria LexA binding sites (top) and the profile emerging from the 93 distinct binding sites located (bottom). Lan = *Listonella anguillarum*; Lpe_CIP = *L. pelagia *CIP 102762T; Val_12G01 = *Vibrio alginolyticus *12G01; Vch_N16961 = *V. cholerae *O1 biovar Eltor str. N16961; Vha_ATCC = *Vibrio harveyi *ATCC BAA-1116; Vha_HY01 = *V. harveyi *HY01; Vme = *Vibrio metschnikovii*; Vmi = *Vibrio mimicus*; Vna_CIP = *Vibrio natriegens *strain CIP 10319; Vpa = *Vibrio parahaemolyticus*; Vpa_RIMD = *V. parahaemolyticus *RIMD 2210633; Vsh_AK1 = *Vibrio shilonii *AK1; Vsp_DAT722 = *Vibrio sp*. DAT722; Vsp_Ex25 = *Vibrio sp*. Ex25; Vvu_CIP754 = *Vibrio vulnificus *CIP 75.4; Vvu_YJ016 = *V. vulnificus *YJ016 (see Additional file 11 for corresponding accession numbers).

We recently identified *E. coli*-like LexA binding sites in the promoter region of *intI1 *integrase genes from mobile integrons and of the *intIA *integrase from the *V. cholerae *superintegron (Figure 2AB). In *V. cholerae *and some of these mobile integrons, the identified LexA boxes partially overlap the -10 element of the *intI *promoter in a classic operator organization. We have shown that expression of *V. cholerae *and *E. coli *pAT674 integrase genes is indeed controlled by the SOS response, leading to heightened rates of integrase-mediated recombination upon SOS induction [25].

To gain insight into the general relevance of this observation, we undertook an exhaustive *in silico *study of integrase regulation by the LexA protein. Using a TBLASTN search (National Center for Biotechnology Information (NCBI); http://blast.ncbi.nlm.nih.gov/), we identified 1,483 homologs of *intIA *in the non-redundant (NR) (971), environmental (ENV) (381) and Whole Genome Shotgun (WGS) (131) subdivisions of the GenBank database. When sufficient data were available (1,103 sequences), the nucleotide sequences corresponding to the first 50 bp of the coding region plus 100 bp upstream of the translation start site (-100, +50) were systematically searched for LexA binding sites. We conducted independent searches for all the 15 LexA binding motifs described to date in the literature [27]. Putative LexA binding sites were detected in 56.6% (624) of the 1,103 sequences for which the (-100,+ 50) region was available (see Additional file 1), with 40 sequences displaying two LexA binding sites in tandem. All the identified LexA binding sites corresponded exclusively to the motif found in *E. coli *and most β/γ-Proteobacteria (Figure 2C). Given that we searched for 14 additional LexA binding motifs and that the sample of integrase sequences contained representatives from the respective clades in which these motifs have been reported, including one α-Proteobacteria species, this strongly suggests that the putative LexA regulation of *intI *genes is restricted to organisms harboring LexA proteins that are able to recognize the β/γ-Proteobacteria. The LexA binding motif of the β/γ-Proteobacteria is markedly divergent from that seen in *E. coli *and the β/γ-Proteobacteria, and it is known to have arisen after the split of the α- and β/γ-Proteobacteria subclasses [45-49]. Hence, it seems very likely that LexA regulation of integrase genes also arose after this evolutionary branching point. When we examined the core 16 bp sequence of the identified *E. coli*-like LexA binding sites, we identified 93 distinct sequences (see Additional file 2). These LexA binding sites presented substantial diversity while maintaining a high level of conservation, as reflected in their joint information content logo (Figure 2C). Importantly, *E. coli*-like LexA sites were detected in almost all Vibrionaceae superintegrons (Figure 2A), and in all but one of the mobile integron classes (Figure 2B), indicating that putative LexA regulation of *intI *genes is a widespread phenomenon among integrons.

### Predicted LexA binding sites correspond to functional transcriptional-control elements

**Figure 3:**
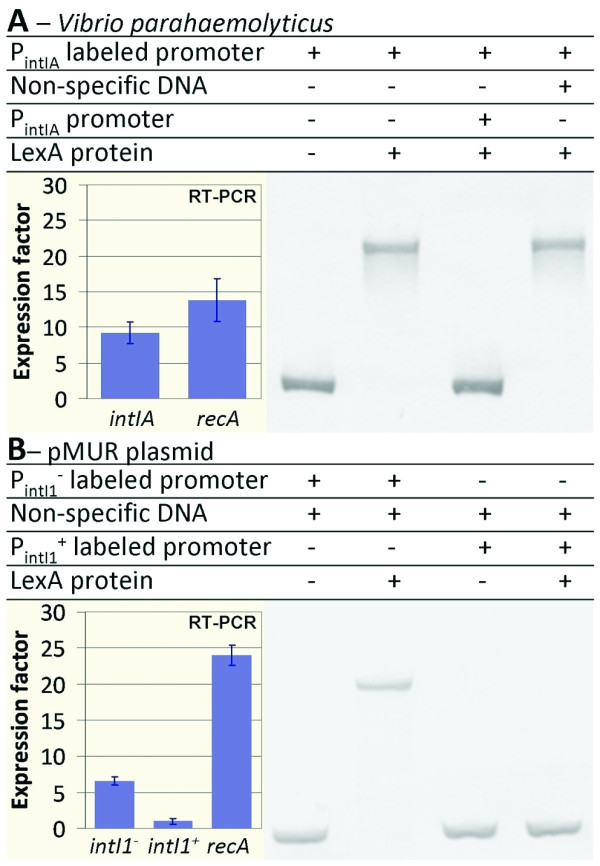
**Electrophoretic mobility-shift assay (EMSA) and quantitative real-time reverse transcription PCR on different *intI *genes and their respective promoters**. **(A) ***Vibrio parahaemolyticus *integron. EMSA of *V. parahaemolyticus intIA *promoter with purified *V. parahaemolyticus *LexA protein. Competitive assays using either non-specific or *P_int _*non-labeled DNA are also shown. The *intA *expression factor was calculated as the ratio of the relative *intA *mRNA concentration in the *V. parahaemolyticus lexA *mutant strain with respect to that in the wild type. (B) *E. coli *pMUR050 integrons. EMSA of pMUR050 *intI1^-^*and *intI1^+^*(containing GGG insertion) promoters with purified *E. coli *LexA protein. The expression factor for both *intI *genes was calculated as the ratio of each relative *intI *mRNA concentration in the *E. coli lexA*-*sulA *mutant strain with respect to that in the wild type. In all cases, the expression factor of *recA *is shown as a control, and all expression factors are the mean value from three independent experiments (each in triplicate).

We have previously shown that LexA regulates the expression of *intI *in *V. cholerae*, and our *in silico *search identified LexA binding sites in the promoter region of *intI *for all sequenced *Vibrio *species (see Additional file 1). To further assess the overall functionality of the *in silico *predicted LexA binding sites, we evaluated integrase LexA regulation in *Vibrio parahaemolyticus *strain ATCC 17802, which harbors a LexA binding site upstream of its *intIA *gene in a genomic context that is substantially different from that of *V. cholerae *(Figure 2A). Using quantitative reverse transcriptase (RT)-PCR, we determined the *intIA *expression level in both the wild-type strain and its *lexA*(Def) derivative (lacking a functional *lexA *gene). We found an expression ratio of 9.28, revealing a strong LexA regulation of the *intIA *gene expression (Figure 3A). Furthermore, electrophoretic mobility-shift assays (EMSA) with purified *V. parahaemolyticus *LexA protein showed that the observed upregulation of *intIA *expression was directly mediated by LexA in this organism (Figure 3A).

In several class 1 integrons, heightened expression of the cassette genes has been shown to rely on a secondary cassette promoter called *P_C2_*, located just upstream of the *intI1 *gene (see Additional file 3). *P_C2 _*is enabled by a GGG insertion (on the top strand) that increases the distance between the -35 box sequence and a sequence resembling the -10 box consensus from 14 to 17 bp, thereby generating a functional σ70 promoter [3,4,50,51]. In all its reported instances, this GGG insertion disrupts a seemingly functional LexA binding site. Therefore, it is likely that the GGG insertion that enables *P_C2 _*should simultaneously abolish integrase regulation by LexA. We tested this hypothesis using the *E. coli *multi-resistant plasmid pMUR050 [52], which provides an ideal background to test this hypothesis because it harbors two integrons with inactivated copies of the *intI1 *gene. The promoter regions of both *intI *genes are almost identical, and differ only in that one (*P_intI1_*^-^) contains a functional LexA binding site in its promoter, whereas the other (*P_intI1_*^+^) presents the aforementioned GGG insertion, disrupting the LexA binding site and enabling the *P_C2 _*promoter (see Additional file 3). As expected, EMSA confirmed that *E. coli *LexA is able to bind the *P_intI1_*^- ^promoter, but that the GGG insertion effectively prevents LexA binding on *P_intI1_*^+ ^(Figure 3B). Furthermore, RT-PCR in wild-type and *lexA*-defective backgrounds confirmed that LexA regulation was only present in the *IntI1^-^*integrase carrying the intact LexA binding site, with a strong deregulation (ratio of 6.55) in the *lexA *mutant (Figure 3B). Thus, the GGG insert not only enables the secondary cassette promoter *P_C2_*, but concomitantly disrupts the LexA binding site of the integrase promoter.

To check whether the GGG insert did in fact lead to the activation of *P_C2 _*and increased cassette expression in the pMUR IntI1^+ ^integrase, we compared RT-PCR expression profiles for the first cassette gene of both pMUR integrons. We found that cassette-gene expression was enhanced in the integron containing the GGG insertion, and that this increase was independent of LexA (data not shown). *In silico *searches for disrupted LexA binding sites found 44 instances of similar GGG inserts in integrons from a wide variety of species (see Additional file 4), all corresponding to class 1 mobile integrons harboring multiple antibiotic-resistance cassettes. Together, these results suggest that LexA regulation may eventually be lost under heavy selection to promote higher basal levels of the antibiotic-resistance transcript.

### Ancestral-state reconstruction of LexA regulation and integrase functionality

The presence of confirmed LexA regulation in *V. cholerae *and *V. parahaemolyticus *superintegrons suggested that SOS control of *intI *genes probably originated very early in their evolutionary history. Likewise, the complete absence of LexA binding motifs different from that of *E. coli *in all the *intI *promoters analyzed in this study indicated that LexA regulation must have been lost in integrons borne by species without LexA, or in which LexA recognizes a divergent motif [27,47]. At the same time, there is ample evidence of extensive (10% to 30%) and independent integrase inactivation across the Bacteria domain, implying that loss of integrase functionality may be an adaptive trait under particular selective pressures [53]. In this respect, the evidence of integrase inactivation in bacterial groups in which it is known that LexA does not recognize the *E. coli *motif [16,54,55], such as the Xanthomonadales, suggests that loss of LexA regulation might be linked to mutational inactivation of the integrase gene.

**Figure 4:**
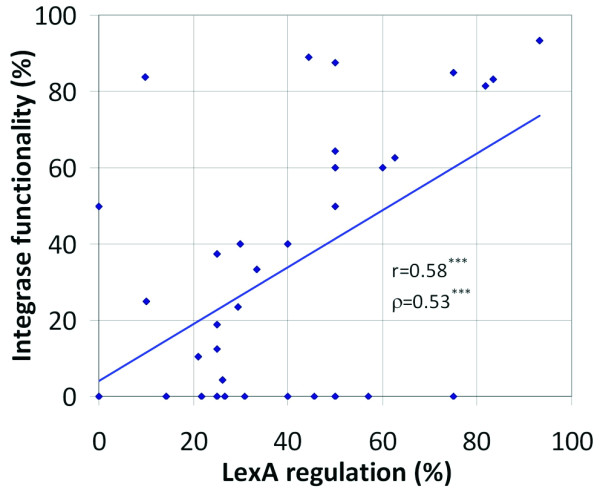
**Correlation between inferred LexA regulation and integron integrase functionality**. The plot was generated from the frequency values for each trait at each reference panel taxon, as derived from reciprocal BLAST mapping **(see **Additional file 14). Pearson and Spearman rank correlations and their respective *P *values were computed in Excel (Microsoft Corp., Redmond, WA, USA). The asterisk rating system is used for correlation *P *values (***P < 0.001). *P *values are relative to two-tailed Student *t*-test on the null hypothesis (no correlation).

To explore this hypothesis, we developed an automated system to assess integrase functionality based on the detection of generic (nonsense and indels) and integrase-specific missense mutations known to inactivate the protein (see Methods). This method was applied to 1,135 *intIA *homologs identified in this work for which sufficient coding sequence was available. Consistent with previous results, we found that a substantial fraction of integrase genes (43%, see Additional file 5) seem to be inactivated [53]. For the 755 *intIA *homologs with sufficient sequence to apply both analyses, the predicted inactivation status for each integrase sequence (active/inactive) was combined with the predicted presence of a LexA binding site in its promoter (-100, +50) region as computed previously. A correlation analysis was carried out to determine the existence of a link between loss of LexA regulation and integrase inactivation. The results of this analysis showed a significant correlation (Pearson *r *= 0.58, Spearman ρ = 0.53; P < 0.001) between both traits (Figure 4), and give credence to the idea that loss of LexA regulation is associated with integrase inactivation.

**Figure 5:**
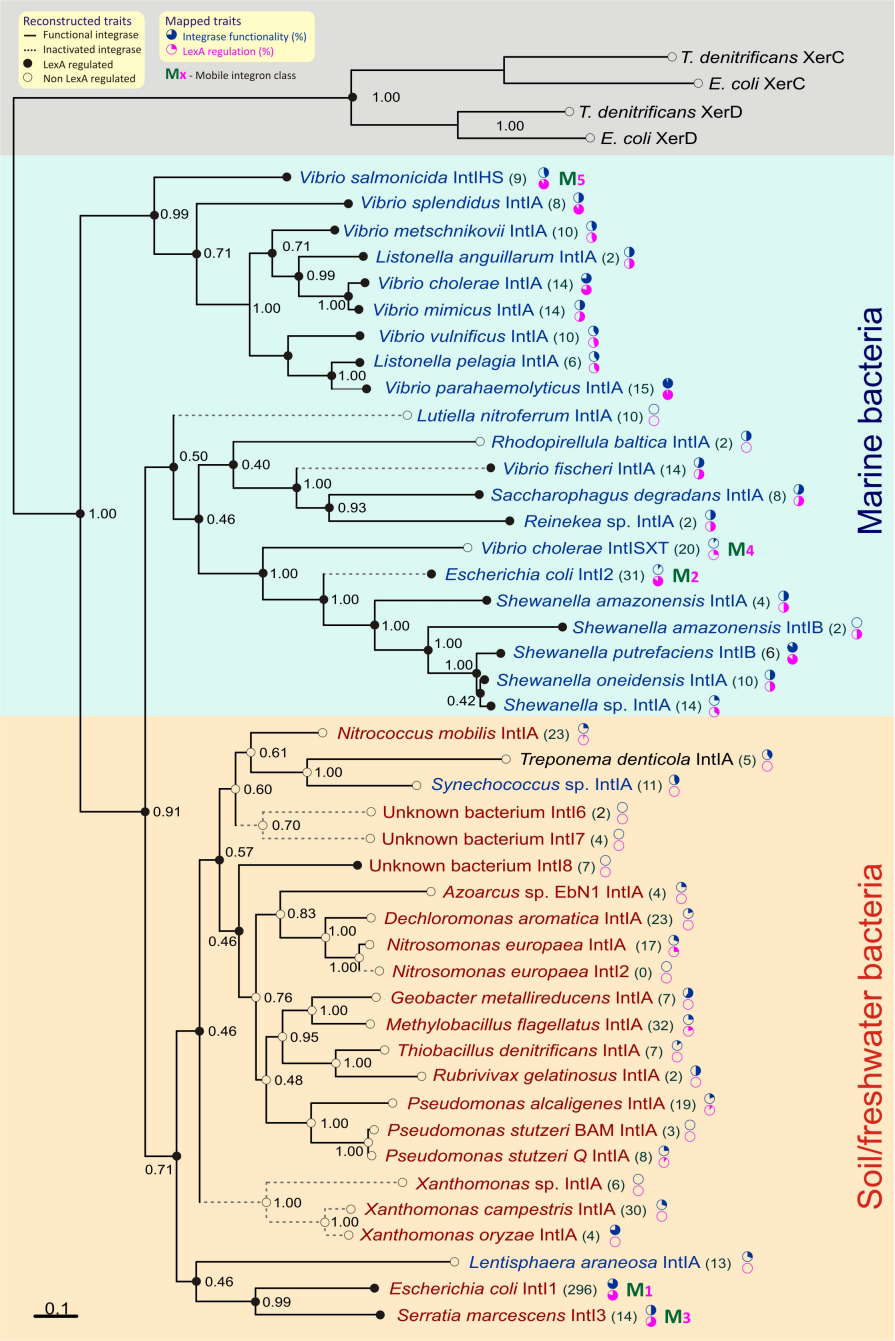
**Phylogenetic tree of IntI protein sequences**. The tree is the majority-rule consensus tree generated by MrBayes. The tree was rooted using the *Escherichia coli *and *Thiobacillus denitrificans *XerCD protein sequences as outgroup. Bayesian posterior probabilities for each branch are displayed at each branching point. Inferred states for phenotypic traits derived from parsimony ancestral-state reconstruction analysis are displayed as follows. Integrase functionality: solid lines on tree branches represent inferred integrase functionality in that branch, and dotted lines indicate non-functionality. LexA regulation: at each taxon and branching point, small filled circles represent inferred presence of LexA regulation, and open circles indicate loss of LexA regulation. For clarity, the results of maximum likelihood reconstruction are not shown (see Additional file 6 and see Additional file 7 for these). The number of sequences mapping to each taxon in the reciprocal BLAST mapping analysis is shown between brackets after the taxon name. Stacked pie charts next to this number indicate the observed percentage of integrase functionality (upper pie) and LexA regulation (lower pie) in all the analyzed integrase sequences mapping to that specific taxon. The *M *letter followed by a subscript number (*M_X_*) legend indicates mobile integron classes (1 to 5). Background colors delineate the main division into marine and soil/freshwater radiations and the XerCD outgroup.

To gain insight into the evolutionary history of this correlation, we generated a phylogenetic tree of 44 representative IntI sequences, and applied ancestral-state reconstruction methods for both phenotypic characters (predicted integrase functionality and LexA regulation). The tree (Figure 5) is in overall agreement with previously published IntI phylogenies [9,53,56]. As in previous phylogenies, two major ecological groups can be outlined on the tree: marine and freshwater/soil bacteria. Chromosomal superintegrons and class 5 mobile integrons borne by marine species form a monophyletic clade that sits at the root of the tree. From this early branch, a second radiation of integrons encompassing both chromosomal integrons and all other mobile integron classes splits neatly into integrons borne by, respectively, marine and soil/freshwater bacteria. In the marine species, class 2 and 4 mobile integrons form a monophyletic cluster with *Shewanella *chromosomal integrons that is also in agreement with previous analyses [57,58]. In the soil/freshwater clade, class 1 and 3 mobile integrases form a distinct group, suggesting an early split from their chromosomal counterparts in the Proteobacteria [59].

Both parsimony and maximum likelihood (ML) reconstructions of the ancestral state for LexA regulation strongly supported the notion that this feature was present in the common ancestor of bacterial integrons. LexA regulation (Figure 5, filled circles) is pervasive among *Vibrio *superintegrons and is also widespread within the marine integron radiation. It is also most likely (0.7 likelihood in ML reconstruction, see Additional file 6) that LexA regulation was present in the ancestor of the soil/freshwater radiation, and has been subsequently lost (Figure 5, open circles) in many of its internal clades. A notable exception to this trend are the class 1 and class 3 integrons, in which LexA regulation is still the norm. Our results thus imply that some particular trait in the environment of both chromosomal superintegrons and mobile integrons must be exerting a considerable selective pressure towards preservation of integrase LexA regulation. In the chromosomal integrons of the Vibrionaceae, the most likely source of this pressure is the stabilization of large integrons, which may include essential genes [15]. In mobile integrons, it seems likely that selection might favor integrons that remain largely inactive, but are capable of generating sharp bursts of recombination activity in times of need for evolutionary innovation.

The reconstruction of ancestral states for inferred integrase functionality is relatively congruent with the hypothesis that the loss of LexA regulation might be associated with integrase inactivation (Figure 5; see Additional file 7). Even though there is testimonial evidence of inactivation (Figure 5, dotted lines), integrases from almost all marine species in the tree were found to be active (Figure 5, solid lines). By contrast, integrase inactivation was found to be monophyletic for two soil/freshwater subgroups, hinting at consistent selective pressure towards inactivation.

### Phylogenetic distribution of predicted LexA regulation and integrase functionality

To further analyze the correlation between integrase LexA regulation and inactivation, we mapped through reciprocal BLAST searches [60] the 755 IntI homologs containing sufficient available sequence to assess both traits against the panel of IntI sequences used to reconstruct the phylogenetic tree. Even though reciprocal BLAST provides only a crude estimate of phylogenetic relationship, this mapping process allowed us to observe the apparent frequencies (Figure 5, pie charts) of both traits in the clusters represented by each tree taxon (Figure 5). Overall, the results of this analysis broadly agree with those of the ancestral-state reconstruction, and give further credence to the idea that loss of LexA regulation is associated with integrase inactivation. Nonetheless, close inspection of these results also reveals a complex pattern of phylogenetic distribution for both traits.

Among marine species, LexA regulation of *intI *genes is clearly prevalent, and loss of LexA regulation is only present in a few instances. One such instance is the *V. cholerae *SXT integrative-conjugative element (ICE), which harbors a class 4 integrase and for which SOS-dependent transfer has been reported through an indirect path involving the phage-like SetR repressor [37,38,61]. In spite of this, mapping results show that five out of the twenty sequences clustering with the *V. cholerae *SXT integrase have predicted LexA binding sites. These sequences belong to mobile integrases in Alteromonadales species that do not have homology with the SetR transcriptional regulator, suggesting that LexA regulation may have been preserved in the absence of SetR-mediated SOS regulation (see Additional file 8). Another exception is *Lutiella nitroferrum*, but the absence of predicted LexA sites is not surprising in a member of the Neisseriaceae, because all the sequenced members of this family lack a *lexA *gene [27]. A similar reasoning applies to another exception, *Rhodopirellula baltica*, because it is known that the LexA of Planctomycetes does not recognize the conventional *E. coli *LexA binding motif [27].

Conversely, loss of LexA regulation seems to be the norm among soil and freshwater species harboring chromosomal integrons. In most cases, this loss of regulation has an obvious explanation. Some families, such as the Nitrosomonadaceae and the Chromatiaceae, simply do not possess any LexA homologs, Thus explaining the absence of any LexA binding sites upstream of their *intI *genes [27]. A similar argument can be made for the Xanthomonadaceae, in which neither of the two identified LexA proteins recognizes the β/γ-Proteobacteria LexA binding motif [54], and for the Spirochetes, the δ-Proteobacteria and the Cyanobacteria, in which LexA also recognizes divergent motifs [48,62,63]. However, reciprocal BLAST mapping indicates that there is residual LexA regulation persisting within several of these groups. The *M. flagellatus *cluster, for instance, has six out of thirty-two mapped sequences with predicted LexA binding sites. Careful examination reveals that, in this and all other cases of residual LexA regulation of soil/freshwater bacterial integrons, regulated integrases turn out to be harbored by a β/γ-Proteobacteria species or originate from environmental samples (see Additional file 9). This strongly suggests that, for the most part, LexA regulation is positively maintained when a suitable genomic background (a compatible *lexA *gene encoding a repressor that recognizes the β/γ-Proteobacteria motif) is available.

Several factors explain partly the absence of LexA binding motifs, other than the β/γ-Proteobacteria motif, regulating integron integrases. An obvious explanation is the lack of evolutionary time to develop such motifs. This is manifestly true for many mobile integrons subject to lateral gene transfer. Indeed, predicted β/γ-Proteobacteria LexA binding sites can still be seen in the mobile integrons harbored by species from distant phyla, such as the Actinobacteria. Integrase inactivation is another mechanism that several groups, such as those of the Xanthomonadales, seem to have evolved to compensate for unregulated integrase expression [16]. Even though this constitutes a general trend (Figure 4) and functionality can be temporarily restored through non-native recombination, the observed correlation is moderate (Pearson *r *= 0.58***). Moreover, integrase functionality has been assayed experimentally in several soil/freshwater chromosomal integrons in which the integrase is clearly not regulated [64,65], suggesting that additional mechanisms must be at play.

Class 1 and 3 mobile integron integrases depart sharply from the trend towards loss of LexA regulation that is seen among soil/freshwater integrons. Reciprocal BLAST mapping supports the results of ancestral-state reconstruction methods, providing ample support for the persistence of LexA regulation in these well-sampled mobile integron classes. In addition, the high percentages of LexA regulation seen in both these integron classes (81% and 64%, respectively) are consistent with high percentages of predicted regulation in the marine mobile integrons of classes 2 and 5 (84% and 89%, respectively; Figure 5). Beyond its fundamental relevance to bacterial adaptation, the high prevalence of predicted LexA regulation of mobile integron integrases has serious clinical implications, as it establishes a generic system for genetic interchange under control of a general stress response shared by a large group of human and animal pathogens. Furthermore, bacterial conjugation has been shown recently to induce the SOS response, triggering integrase-mediated cassette recombination, in recipient bacteria [66]. In this setting, it is important to note that integron cassettes encoding resistance to several antibiotics known to induce the SOS response, such as trimethoprim, quinolones and β-lactams, are common today [5,67]. This suggests that the indirect triggering effect of these antibiotics on the capture of resistance cassettes may have resulted in a very efficient selection mechanism.

A less obvious consequence of integrase SOS regulation in clinically relevant mobile integrons is its repercussion on antibiotic-resistance policies. Current policies rely largely on the detrimental effects that most resistance mechanisms inflict on bacteria, which eventually lead to loss of resistance genes in the absence of antibiotic exposure [68]. Because most cassettes are promoterless, the most ancient cassettes (located at the distal part of the integron) are subject to severe polar effects, leading to rare or non-existent protein products (Figure 1) [4]. In this context, the incorporation of SOS regulation in integrons puts forward a mechanism by which antibiotic-resistance genes and other useful adaptations can be silently set aside, while current adaptive traits are maintained. In time of stress, such as exposure to antibiotics, the relevant resistance cassettes can be called upon by integrase-mediated translocation, and thus selected for only when their expression is required. Furthermore, the cassette genes that have been temporarily relegated to distal positions in integrons may also sustain increased evolution rates, generating a substantial pool of variability from which to draw on when the appropriate selective pressures resurface [69].

Reciprocal BLAST mapping also shows that predicted integrase inactivation is very common among soil/freshwater bacteria, coinciding with a prevailing loss of putative LexA regulation. Nonetheless, predicted integrase inactivation is also relatively common in marine species. Even though the predicted integrase inactivation correlates well with reduced LexA regulation (Figure 4), there are notable outliers to this trend in both radiations. For instance, among mobile class 5 integrons, only 44% of mapped integrases seem to be functional, despite predicted LexA regulation in 89% of them. The opposite is also true; many mobile integrons with putatively functional integrases have disrupted, absent or non-native LexA binding sites. This suggests that lack of LexA regulation can be tolerated or selected for when it provides adaptive benefit. We have shown here that in some mobile class 1 integrons, the LexA binding site has been disrupted by a GGG insertion that drastically increases the expression of antibiotic-resistance cassettes (Figure 3). In a similar vein, it seems likely that sustained integrase activity (with its associated shuffling of gene cassettes [21,25]) must be preferable to permanent inactivation under the shifting selective environments associated with clinical environments and mobile integrons. This would explain why integrase inactivation is not seen as frequently in mobile integron classes associated with clinical settings, in spite of their dissemination into bacterial species that do not harbor a *lexA *gene capable of regulating the preset LexA binding site.

Overall, however, the pattern of integrase inactivation is broadly in agreement with that reported previously [53]. In fact, we found a higher proportion (46%) of inactivated IntI proteins than that reported previously [53], indicating that integrase inactivation is a pervasive phenomenon and typically correlated with loss of LexA regulation. Hence, our findings suggest that putative integrase inactivation is the main mechanism evolved to deal with lack of LexA regulation, but it seems likely that other factors must provide heightened tolerance to unregulated integrase activity in soil/freshwater bacteria. Smaller integron sizes and lessened integrase activity may both contribute to make unregulated integrase expression more tolerable, but regulation by an alternative transcription factor is an obvious possibility that needs to be carefully explored. This is particularly true because most integrase functionality assays have been carried out in a non-native context [64,65] and may thus have missed regulatory effects. The quest to define precisely the multiple mechanisms behind this adaption is an important goal, because the lack of a mechanism to mitigate the effects of integrase activity upon loss of LexA regulation may well lie at the root of the intriguing absence of chromosomal integrons from many bacterial phyla [53].

## Conclusions

The results presented here illustrate the extent of SOS regulation of integron integrases, and provide several important clues to the evolution of this regulation and to the evolution of bacterial integrons. The combination of *in silico *and *in vivo *assays allows us to conclude that LexA regulation was probably present in the primordial integron and that its loss may be linked to a number of factors, including inactivation of the integrase gene and enhancement of resistance cassettes expression. Our findings have important clinical implications for the evolution of antibiotic resistance, and suggest that the emergence of mechanisms to palliate unregulated integrase expression may provide an explanation for the uneven distribution of integrons across the Bacteria domain.

## Methods

### Data mining and preprocessing

A custom set of scripts was developed in BioPhyton to search for *intI *homologs on NCBI GenBank databases (NR, ENV and WGS). The scripts retrieved and re-annotated both the *intI *coding sequences and their corresponding upstream sequences. The scripts used the whole VchIntIA protein sequence (AAC38424) and its IntI specific domain [70] (positions 186 to 245 in VchIntIA) as a query for a TBLASTN search. To limit the number of false positives, a cut-off e-value of 10^-5 ^was set, and only sequences matching both queries were retrieved.

TBLASTN results were used to identify frameshift and deletion events of up to 100 bp. Larger events where not considered. The nucleotide sequences spanning the full length of the processed hits and 1 kb upstream of the hit start were recovered. Conceptual translations of these sequences (corrected for frameshift when necessary) were then used to search a curated reference panel using BLASTP. The reference panel comprised 43 phylogenetically diverse IntI proteins, phage integrases and XerCD recombinases. The reference sequence of the best reciprocal hit was used to consistently re-annotate the start and stop points of all retrieved sequences, thereby allowing homogenization of the dataset and efficient detection of in-frame premature stop codons. Sequences with a best reciprocal hit not belonging to the IntI family (that is, phage integrases and XerCD recombinases) were removed from further analysis. Similarly, all IntI homologs lacking a significant amount of coding sequence at both ends of the predicted coding region (+30 bp downstream of the start codon and -90 bp upstream of stop codon) were also removed from further analysis. Duplicates resulting from the use of partially redundant databases were removed, defining duplicates as two sequences having the same sequences, coordinates and NCBI taxonomical assignment, and the same strain or plasmid number when applicable. The final annotated dataset comprised 1,483 sequences, and is available online as supplementary material in both GenBank (.GBK) and spreadsheet-compatible (.XLS) format (see Additional file 10, see Additional file 11).

### Assessment of protein functionality

Integrase functionality was assessed systematically using a custom rule-based system operating on aligned IntI sequences. To generate functional rules to detect inactivation, we analyzed published structural and mutational studies of both the chromosomal *V. cholerae *IntI4 and the mobile IntI1 integron integrases [22,70-74]. From this analysis, we identified a list of five essential residues in the catalytic site that cannot be mutated (R135, K160, H267, R270, H293, Y302; positions relative to the *V. cholerae *IntIA sequence), and eight residues essential for binding, for which only a limited range of substitutions is likely to be tolerated (L202 (→LIVM), P203 (→PST), K209, Y210 (→YFWH), P211 (→PRQ), R239 (→KRH), H240 (→KRH), H241 (→KRH); positions again relative to the *V. cholerae *IntIA sequence).

A multiple alignment of all IntI sequences in the reference panel was generated using MUSCLE software http://www.drive5.com/muscle/ with an opening gap penalty of -20, and otherwise standard parameters [75]. This alignment was used to propagate the functional rules defined on the VchIntIA sequence towards the reference panel IntI sequences. The consistency of this propagation was reviewed manually. Pairwise alignments of all the TBLASTN identified homologs with their corresponding best hits were used to further propagate the functional model and allow a decision on whether each particular protein should be considered functional. IntI sequences containing an internal stop, a frameshift and/or any number of inactivating mutations were tagged as 'non-functional'. If either the start or stop of sequence was unavailable (see above), the functionality of the corresponding protein was tagged as 'unknown'. Otherwise, the protein was considered functional by default.

The automated rule-based system was evaluated against a reference set of integron integrase sequences for which activity has been experimentally assessed [64,65,76-80]. This reference set encompasses active and inactive integrases from both marine and soil/freshwater chromosomal integrons, and class 1, 2 and 3 mobile integrons. The rule-based system was able to correctly predict integrase activity in all these cases. In addition, it also detected all indels, frameshift and nonsense mutations that have been reported previously in independent studies as leading to integrase inactivation [16,53].

### *In silico *searches for LexA binding sites

The presence of LexA binding sites on all the retrieved *intI *homolog sequences was assessed by scanning them using xFITOM http://compbio.umbc.edu/2280/, a generic program for binding site search in genomic sequences [81,82]. Searches were conducted using the *R_i _*index [83] and a motif-normalized threshold as reported previously [84]. Identified sites were considered 'w/functional box' if located within -100 or +50 bp of the re-annotated *intI *start codon. When the sequence in the specified range was not fully available, this feature was tagged as 'unknown'. Searches were conducted using the 15 different LexA binding motifs reported to date [27], which include those of largely sampled phylogenetic groups, such as the Firmicutes, the Actinobacteria, the Cyanobacteira or the Alpha Proteobacteria [62,85-87]. We also identified, and specifically searched for, a particular motif consisting of a LexA binding site inactivated by the insertion of a GGG triplet. These sites are referred to as 'broken', and were categorized as 'without functional box'. The results of integrase functionality and LexA binding site searches are fully annotated on the main dataset files (see Additional file 10, see Additional file 11).

### Phylogenetic analyses

Alignments of the reference-panel protein sequences were carried out using a combined procedure to improve alignment quality as described previously [88]. Protein sequences were first aligned with CLUSTALW (version1.83; http://www.ebi.ac.uk/Tools/msa/clustalw2/[89] using Gonnet matrices and default [10], twenty-five and five gap-opening penalties for the multiple alignment stage, thus generating three different alignments. These three different alignments, together with a local alignment generated by the T-COFFEE Lalign method, were integrated as libraries into T-COFFEE (version 1.37; http://www.ebi.ac.uk/Tools/msa/tcoffee/[90] for optimization. The optimized alignment was then processed with Gblocks (version 0.91b; http://molevol.cmima.csic.es/castresana/Gblocks.html[91] with the half-gaps setting and otherwise default parameters to select conserved positions and discard poorly aligned and phylogenetically unreliable information. Phylogenetic analyses were then carried out using MrBayes (version 3.1.1; http://mrbayes.csit.fsu.edu/ and PHYML version 2.4.1; http://code.google.com/p/phyml/[92] for Bayesian inference of tree topologies as reported previously [88]. A mixed four-category γ distributed rate plus proportion of invariable sites model [invgamma] was applied and its parameters were estimated independently by the program. Eight independent MrBayes Metropolis-Coupled Markov Chain Monte Carlo runs were carried out with four independent chains for 10^6 ^generations. The resulting phylogenetic trees were plotted with TreeView (version 1.6.6; http://taxonomy.zoology.gla.ac.uk/rod/treeview.html[93] and edited for presentation using CorelDraw Graphic Suite (version 12; Corel Corp., Fremont, CA, USA).

Ancestral-state reconstruction was conducted with the Mesquite ancestral-state reconstruction package (Mesquite Software Inc., Austin, TX, USA) [94] using the majority-rule consensus tree generated by MrBayes. The results of *in silico *searches for LexA binding sites were mapped into a discrete (1/0/?) character for each taxon of the tree. Reconstruction of LexA binding site presence was first carried out using the ML reconstruction method [95,96] and the AsymmMk model (Asymmetrical Markov k-state two-parameter model), estimating asymmetric rates of change between characters. The estimated rates (0.145 forward, 0.813 backward) were then converted into parsimony steps by direct inversion (6.89, 1.23), and used to generate the step matrix for parsimony reconstruction [97]. The results from *in silico *integrase functionality assessment were also mapped into a discrete (1/0/?) character for each taxon. Ancestral-state reconstruction for this character was carried out using both an ordered parsimony model and AsymmMk-based maximum-likelihood model. The results of both reconstruction methods were broadly in agreement (see Additional file 7), but for clarity, only parsimony results are superimposed on Figure 5.

### EMSA

The *V. parahaemolyticus *and *E. coli lexA *genes were amplified using suitable primers (see Additional file 12) and cloned into a pET15b vector (see Additional file 13). Overexpression and purification of the corresponding LexA protein was performed as described previously for other LexA proteins [84]. Each DNA probe was constructed using two complementary 100 bp synthetic oligonucleotides (see Additional file 12). EMSA experiments were performed as described previously [84], using 80 nmol/l *V. parahaemolyticus *LexA or 200 nmol/l of *E.coli *LexA protein and 20 ng of each DIG-marked DNA probe in the binding mixture. For EMSA competitive assays, 200 fold of either specific or non-specific non-labeled DNA was added to the binding mixture. In all cases, samples were loaded onto 6% non-denaturing Tris-glycine polyacrylamide gels. Digoxigenin-labeled DNA-protein complexes were detected using the manufacturer's protocol (Roche Applied Science, Indianapolis, IN, USA).

### RNA extraction and RT-PCR

RT-PCR experiments were performed (Titan One Tube RT-PCR System; Roche) with suitable oligonucleotides (see Additional file 12 for list), following the manufacturer's instructions. Real-time quantitative RT-PCR analysis of total RNA was carried out in a PCR system, (LightCycler; Roche), using a commercial kit (LCRNA Master SYBR Green I Kitl Roche) according to the manufacturer's instructions. Transcription of pMUR050 *intID1 *and *intID2 *genes (under control of *P_intI1_*^- ^and *P_intI1_*^+^, respectively) was determined in wild-type *E. coli *K12 and in a *lexA*-defective strain (UA6189). Both strains contained either the pUA1105 (*intA1*) or the pUA1106 (*intA2*) plasmid. Expression of the *V. parahaemolyticus intI *gene was tested in the ATCC17802 wild-type strain and in a *lexA*-defective strain (UA10001) (see Additional file 13). In both cases, expression of the *recA *gene was used as the positive control, and the mRNA concentration for each gene was normalized to that of the housekeeping *dxs *gene. The expression factor was calculated as the ratio of the relative mRNA concentration for each gene in the corresponding *lexA *mutant strain with respect to that in the wild type. In each case, the mean value from three independent experiments (each in triplicate) was calculated. Strains UA6189 and UA10001 were constructed, respectively, using the Lambda-Red recombinase system [98] or the marker exchange procedure, as described previously [99].

## Competing interests

The authors declare that they have no competing interests.

## Authors' contributions

GC implemented the functionality assessment method and carried out data mining, preprocessing and statistical analysis. NSA performed protein purification, RT-PCR and mobility-shift assays. SC and JB designed and directed the *in vitro *and *in vivo *studies and provided expertise on LexA binding motifs. EG, SDR and MCP coordinated *in vitro *and *in vivo *analyses and provided expertise on integrase expression. BGZ provided the pMUR plasmid and participated in coordination. GC, NSA, DM and IE conceived of the study and participated in its design and coordination. IE developed the site-search method, carried out phylogenetic reconstruction and ancestral-state reconstruction, and directed the *in silico *and statistical analyses. DM and GC developed the functionality assessment method. DM and IE coordinated and directed this work and drafted the manuscript. All authors read and approved the final manuscript.

## Funding

This work wmas supported by grants from the Ministère de la Recherche et de l'Enseignement supérieur, the Conseil Régional du Limousin, the Fondation pour la Recherche Médicale (FRM) and from the Institut National de la Santé et de la Recherche Médicale (Inserm) for the Ploy laboratory; by the Institut Pasteur, the Centre National de la Recherche Scientifique (CNRS URA 2171), the FRM and the EU (NoE EuroPathoGenomics, LSHB-CT-2005-512061), for the Mazel laboratory; and by grants BFU2008-01078/BMC from the Ministerio de Ciencia e Innovación de España and 2009SGR-1106 from the Generalitat de Catalunya, for the Barbé laboratory. NSA was supported by the Fundació Cellex at the Erill laboratory.

## Supplementary Material

Additional file 1 **Identified LexA binding sites in the promoter region (-100, +50 of the start codon) of integrase homologs from the WGS, NR and ENV NCBI databases**. Click here for file

Additional file 2 **List of the 93 unique, distinct LexA binding sites identified in this work**. Click here for file

Additional file 3 **(A) Schematic representation of the pMUR050 plasmid, showing (bold) the location of the two *intI1 *homologs**. **(B) **Schematic representation of the promoter region of both *intI1 *homologs, showing the organization of the *P_intI1_*^- ^and *P_intI1_*^+ ^promoters, the standard cassette promoter (*P_C _*and the secondary cassette promoter (*P_C2_*) enabled by the GGG insertion. For both genes, promoter elements are also mapped into their corresponding sequence fragments. Red boxes depict LexA binding sites, black boxes outline the -35 and -10 elements of the *P_intI1 _*promoter, and green boxes depict the secondary *P_C2 _*promoter. Click here for file

Additional file 4 **List of the 45 LexA binding sites presenting GGG disruption identified in this work**. Click here for file

Additional file 5 **Predicted functionality status for the 1,135 *intI *homolog sequences for which sufficient coding sequence was available to determine inactivation using the *in silico *method reported in this work**. Click here for file

Additional file 6 **Phylogenetic tree of IntI protein sequences showing the maximum likelihood ancestral-state reconstruction of LexA regulation, as inferred from *in silico *analyses, using an asymmetric two-state Markov model (AsymmMk) in Mesquite **[94]. The tree is the majority-rule consensus tree generated by MrBayes, and was rooted using the *Escherichia coli *and *Thiobacillus denitrificans *XerCD protein sequences as outgroup. At each taxon and branching point, pie-filled circles indicate the likelihood of LexA regulation at each node, with a completely filled circle indicating certainty of LexA regulation, and a completely open circle indicating certainty of lack of LexA regulation. Taxon name colors indicate the natural habitat of each organism (blue for marine, green for soil/freshwater, black for ambiguous) or their pertaining to the outgroup (red). Azo = *Azoarcus *sp. EbN1; Dar = *Dechloromonas aromatica*; Eco = *E. coli*; Gme = *Geobacter metallireducens*; Lan = *Listonella anguillarum*; Lar = *Lentisphaera araneosa*; Lni = *Lutiella nitroferrum*; Lpe = *Listonella pelagia*; Mfl = *Methylobacillus flagellatus*; Neu = *Nitrosomonas europaea*; Nmo = *Nitrococcus mobilis*; Pal = *Pseudomonas alcaligenes*; Pme = *Pseudomonas mendocina*; Ppr = *Photobacterium profundum*; PstuBA = *Pseudomonas stutzeri *BAM; PstuQ = *Pseudomonas stutzeri *Q; Rei = *Reinekea *sp.; Rba = *Rhodopirellula baltica*; Rge = *Rubrivivax gelatinosus*; Sde = *Saccharophagus degradans*; Sam = *Shewanella amazonensis*; Ssp = *Shewanella *sp. MR-7; Son = *Shewanella oneidensis*; Spu = *Shewanella putrefaciens*; SynSp = *Synechococcus *sp; Tden = *Treponema denticola*; Tde = *Thiobacillus denitrificans*; Vch = *Vibrio cholerae*; Vfi = *Vibrio fischeri*; Vme = *Vibrio metschnikovii*; Vmi = *Vibrio mimicus*; Vpa = *Vibrio parahaemolyticus*; Vsp = *Vibrio splendidus*; Vvu = *Vibrio vulnificus*; Xca = *Xanthomonas campestris*; Xor = *Xanthomonas oryzae*; Xsp = *Xanthomonas *sp. Click here for file

Additional file 7 **Phylogenetic tree of IntI protein sequences showing the maximum likelihood ancestral-state reconstruction of integrase functionality, as inferred from *in silico *analyses, using an asymmetrical two-state Markov model (AsymmMk) in Mesquite **[94]. The tree is the majority-rule consensus tree generated by MrBayes, and was rooted using the *Escherichia coli *and *Thiobacillus denitrificans *XerCD protein sequences as outgroup. At each taxon and branching point, pie-filled circles indicate the likelihood of integrase functionality at each node, with a completely filled circle indicating certainty of integrase functionality and a completely open circle indicating certainty of integrase inactivation. Taxon name colors indicate the natural habitat of each organism (blue for marine, green for soil/freshwater, black for ambiguous) or their pertaining to the outgroup (red). Azo = *Azoarcus *sp. EbN1; Dar = *Dechloromonas aromatica*; Eco = *Escherichia coli*; Gme = *Geobacter metallireducens*; Lan = *Listonella anguillarum*; Lar = *Lentisphaera araneosa*; Lni = *Lutiella nitroferrum*; Lpe = *Listonella pelagia*; Mfl = *Methylobacillus flagellatus*; Neu = *Nitrosomonas europaea*; Nmo = *Nitrococcus mobilis*; Pal = *Pseudomonas alcaligenes*; Pme = *Pseudomonas mendocina*; Ppr = *Photobacterium profundum*; PstuBA = *Pseudomonas stutzeri *BAM; PstuQ = *Pseudomonas stutzeri *Q; Rei = *Reinekea *sp.; Rba = *Rhodopirellula baltica*; Rge = *Rubrivivax gelatinosus*; Sde = *Saccharophagus degradans*; Sam = *Shewanella amazonensis*; Ssp = *Shewanella *sp. MR-7; Son = *Shewanella oneidensis*; Spu = *Shewanella putrefaciens*; SynSp = *Synechococcus *sp; Tden = *Treponema denticola*; Tde = *Thiobacillus denitrificans*; Vch = *Vibrio cholerae*; Vfi = *Vibrio fischeri*; Vme = *Vibrio metschnikovii*; Vmi = *Vibrio mimicus*; Vpa = *Vibrio parahaemolyticus*; Vsp = *Vibrio splendidus*; Vvu = *Vibrio vulnificus*; Xca = *Xanthomonas campestris*; Xor = *Xanthomonas oryzae*; Xsp = *Xanthomonas *sp. Click here for file

Additional file 8 **List of sequences mapping to the VchIntISXT (AAK95987) taxon according to reciprocal BLAST results**. Sequences belonging to the Alteromonadales order are shown yellow. Click here for file

Additional file 9 **Sequences from soil/freshwater bacteria clusters showing evidence of residual LexA regulation**. Sequences with identified LexA binding sites are shown in yellow. Click here for file

Additional file 10 **Complete and fully annotated set of IntI homologs identified in this work in GenBank format**. Click here for file

Additional file 11 **Complete and fully annotated set of IntI homologs identified in this work in Excel (XLS) format**. Click here for file

Additional file 12 **Oligonucleotides used in this work**. Click here for file

Additional file 13 **Strains and plasmids used in this work**. Click here for file

Additional file 14 **Statistical summary of reciprocal BLAST mapping results with regard to the two *in silico *predicted traits analyzed here: LexA regulation and integrase functionality**. Click here for file
