# Supplementary figures and images for: Prevalence of SOS-mediated control of integron integrase expression as an adaptive trait of chromosomal and mobile integrons
Source: Mob DNA. 2011 Apr 30;2:6. doi: 10.1186/1759-8753-2-6 (PMC3108266; doi:10.1186/1759-8753-2-6)

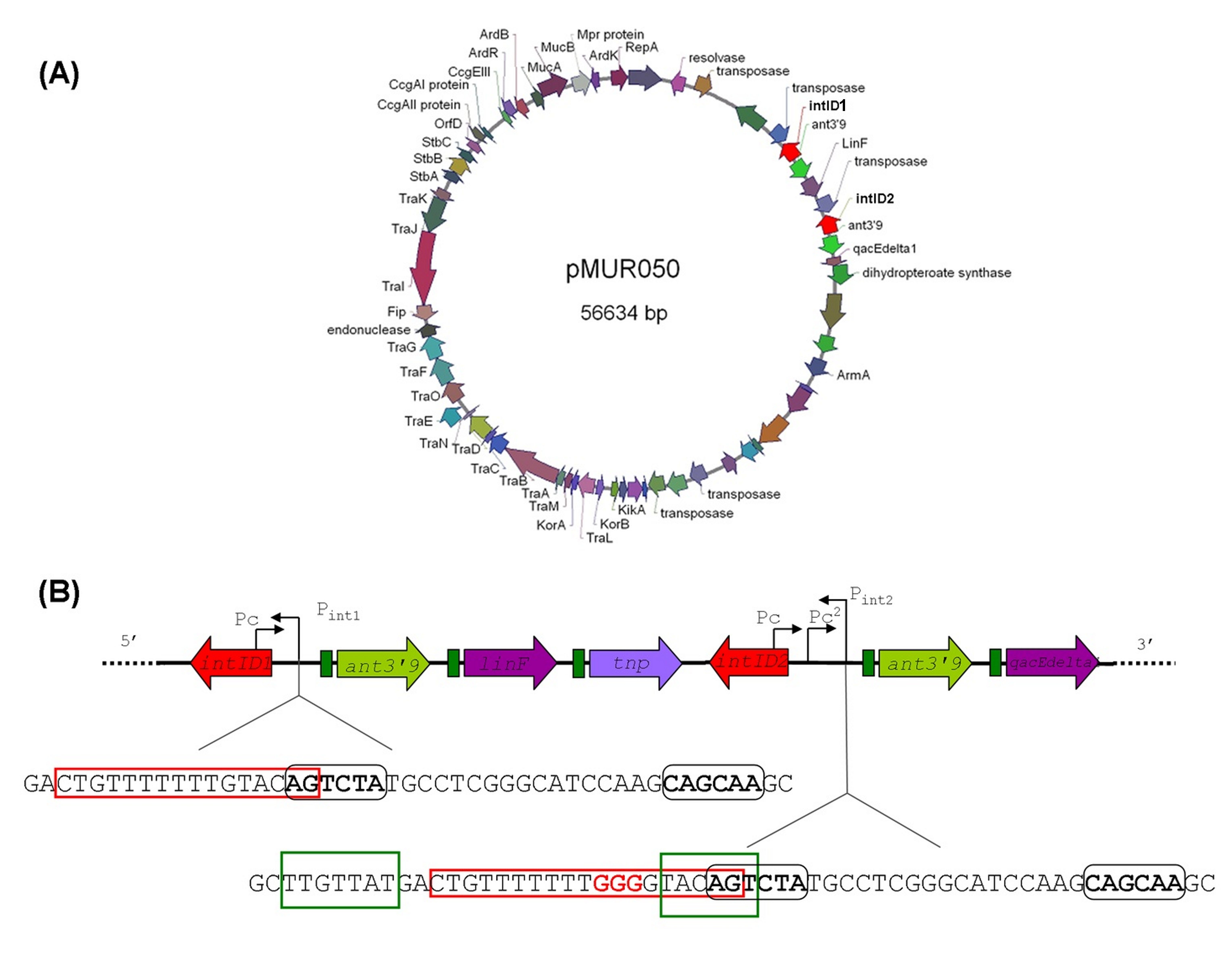

Supplement: Additional file 3 — (A) Schematic representation of the pMUR050 plasmid, showing (bold) the location of the two intI1 homologs. (B) Schematic representation of the promoter region of both intI1 homologs, showing the organization of the PintI1- and PintI1+ promoters, the standard cassette promoter (PC and the secondary cassette promoter (PC2) enabled by the GGG insertion. For both genes, promoter elements are also mapped into their corresponding sequence fragments. Red boxes depict LexA binding sites, black boxes outline the -35 and -10 elements of the PintI1 promoter, and green boxes depict the secondary PC2 promoter. [file 1759-8753-2-6-S3.JPEG]

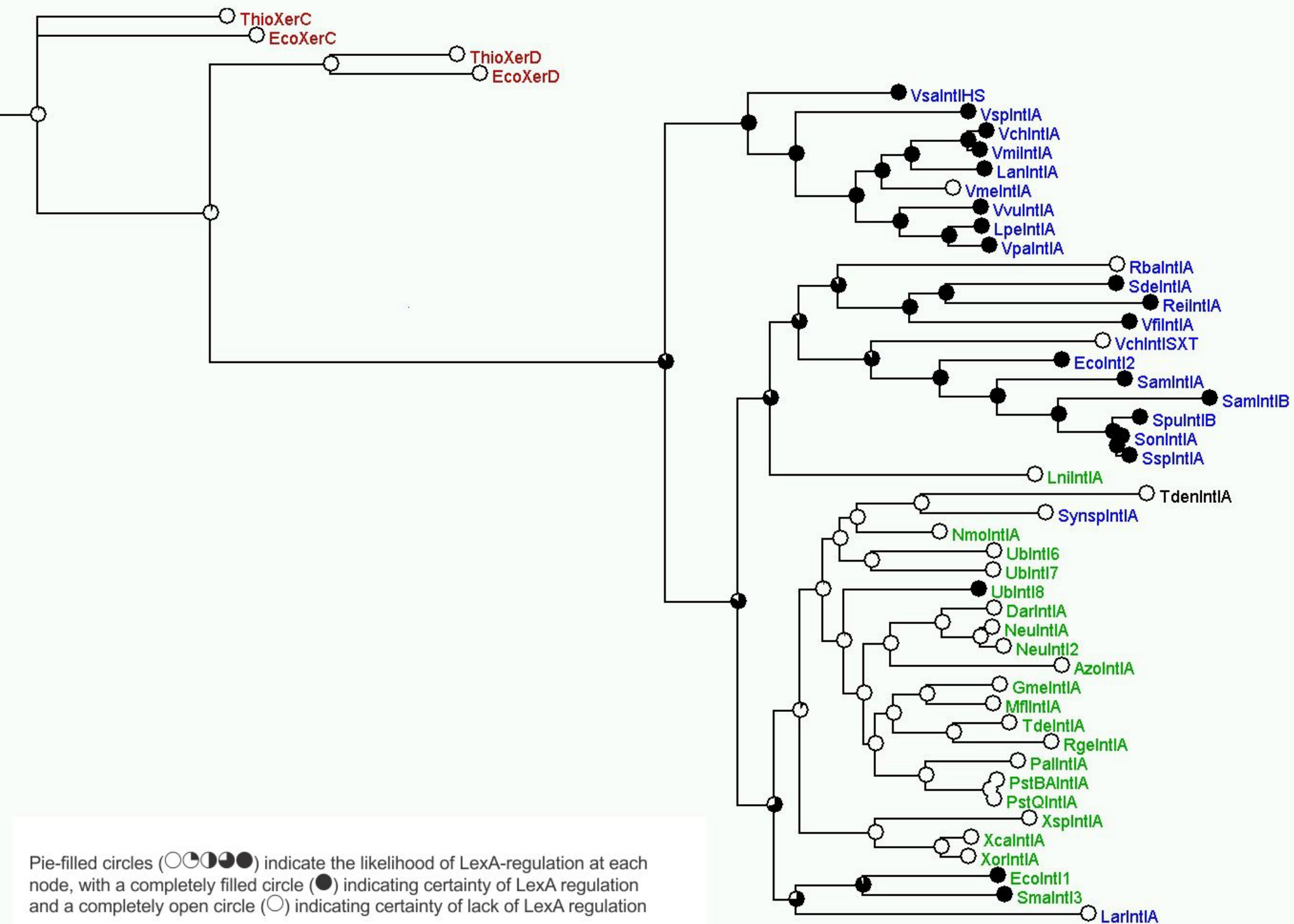

Supplement: Additional file 6 — Phylogenetic tree of IntI protein sequences showing the maximum likelihood ancestral-state reconstruction of LexA regulation, as inferred from in silico analyses, using an asymmetric two-state Markov model (AsymmMk) in Mesquite [94]. The tree is the majority-rule consensus tree generated by MrBayes, and was rooted using the Escherichia coli and Thiobacillus denitrificans XerCD protein sequences as outgroup. At each taxon and branching point, pie-filled circles indicate the likelihood of LexA regulation at each node, with a completely filled circle indicating certainty of LexA regulation, and a completely open circle indicating certainty of lack of LexA regulation. Taxon name colors indicate the natural habitat of each organism (blue for marine, green for soil/freshwater, black for ambiguous) or their pertaining to the outgroup (red). Azo = Azoarcus sp. EbN1; Dar = Dechloromonas aromatica; Eco = E. coli; Gme = Geobacter metallireducens; Lan = Listonella anguillarum; Lar = Lentisphaera araneosa; Lni = Lutiella nitroferrum; Lpe = Listonella pelagia; Mfl = Methylobacillus flagellatus; Neu = Nitrosomonas europaea; Nmo = Nitrococcus mobilis; Pal = Pseudomonas alcaligenes; Pme = Pseudomonas mendocina; Ppr = Photobacterium profundum; PstuBA = Pseudomonas stutzeri BAM; PstuQ = Pseudomonas stutzeri Q; Rei = Reinekea sp.; Rba = Rhodopirellula baltica; Rge = Rubrivivax gelatinosus; Sde = Saccharophagus degradans; Sam = Shewanella amazonensis; Ssp = Shewanella sp. MR-7; Son = Shewanella oneidensis; Spu = Shewanella putrefaciens; SynSp = Synechococcus sp; Tden = Treponema denticola; Tde = Thiobacillus denitrificans; Vch = Vibrio cholerae; Vfi = Vibrio fischeri; Vme = Vibrio metschnikovii; Vmi = Vibrio mimicus; Vpa = Vibrio parahaemolyticus; Vsp = Vibrio splendidus; Vvu = Vibrio vulnificus; Xca = Xanthomonas campestris; Xor = Xanthomonas oryzae; Xsp = Xanthomonas sp. [file 1759-8753-2-6-S6.PDF]
