# Supplementary material for: Prevalence of SOS-mediated control of integron integrase expression as an adaptive trait of chromosomal and mobile integrons
Source: Mob DNA. 2011 Apr 30;2:6. doi: 10.1186/1759-8753-2-6 (PMC3108266; doi:10.1186/1759-8753-2-6)
Supplement: Additional file 7 — Phylogenetic tree of IntI protein sequences showing the maximum likelihood ancestral-state reconstruction of integrase functionality, as inferred from in silico analyses, using an asymmetrical two-state Markov model (AsymmMk) in Mesquite [94]. The tree is the majority-rule consensus tree generated by MrBayes, and was rooted using the Escherichia coli and Thiobacillus denitrificans XerCD protein sequences as outgroup. At each taxon and branching point, pie-filled circles indicate the likelihood of integrase functionality at each node, with a completely filled circle indicating certainty of integrase functionality and a completely open circle indicating certainty of integrase inactivation. Taxon name colors indicate the natural habitat of each organism (blue for marine, green for soil/freshwater, black for ambiguous) or their pertaining to the outgroup (red). Azo = Azoarcus sp. EbN1; Dar = Dechloromonas aromatica; Eco = Escherichia coli; Gme = Geobacter metallireducens; Lan = Listonella anguillarum; Lar = Lentisphaera araneosa; Lni = Lutiella nitroferrum; Lpe = Listonella pelagia; Mfl = Methylobacillus flagellatus; Neu = Nitrosomonas europaea; Nmo = Nitrococcus mobilis; Pal = Pseudomonas alcaligenes; Pme = Pseudomonas mendocina; Ppr = Photobacterium profundum; PstuBA = Pseudomonas stutzeri BAM; PstuQ = Pseudomonas stutzeri Q; Rei = Reinekea sp.; Rba = Rhodopirellula baltica; Rge = Rubrivivax gelatinosus; Sde = Saccharophagus degradans; Sam = Shewanella amazonensis; Ssp = Shewanella sp. MR-7; Son = Shewanella oneidensis; Spu = Shewanella putrefaciens; SynSp = Synechococcus sp; Tden = Treponema denticola; Tde = Thiobacillus denitrificans; Vch = Vibrio cholerae; Vfi = Vibrio fischeri; Vme = Vibrio metschnikovii; Vmi = Vibrio mimicus; Vpa = Vibrio parahaemolyticus; Vsp = Vibrio splendidus; Vvu = Vibrio vulnificus; Xca = Xanthomonas campestris; Xor = Xanthomonas oryzae; Xsp = Xanthomonas sp. [file 1759-8753-2-6-S7.PDF]

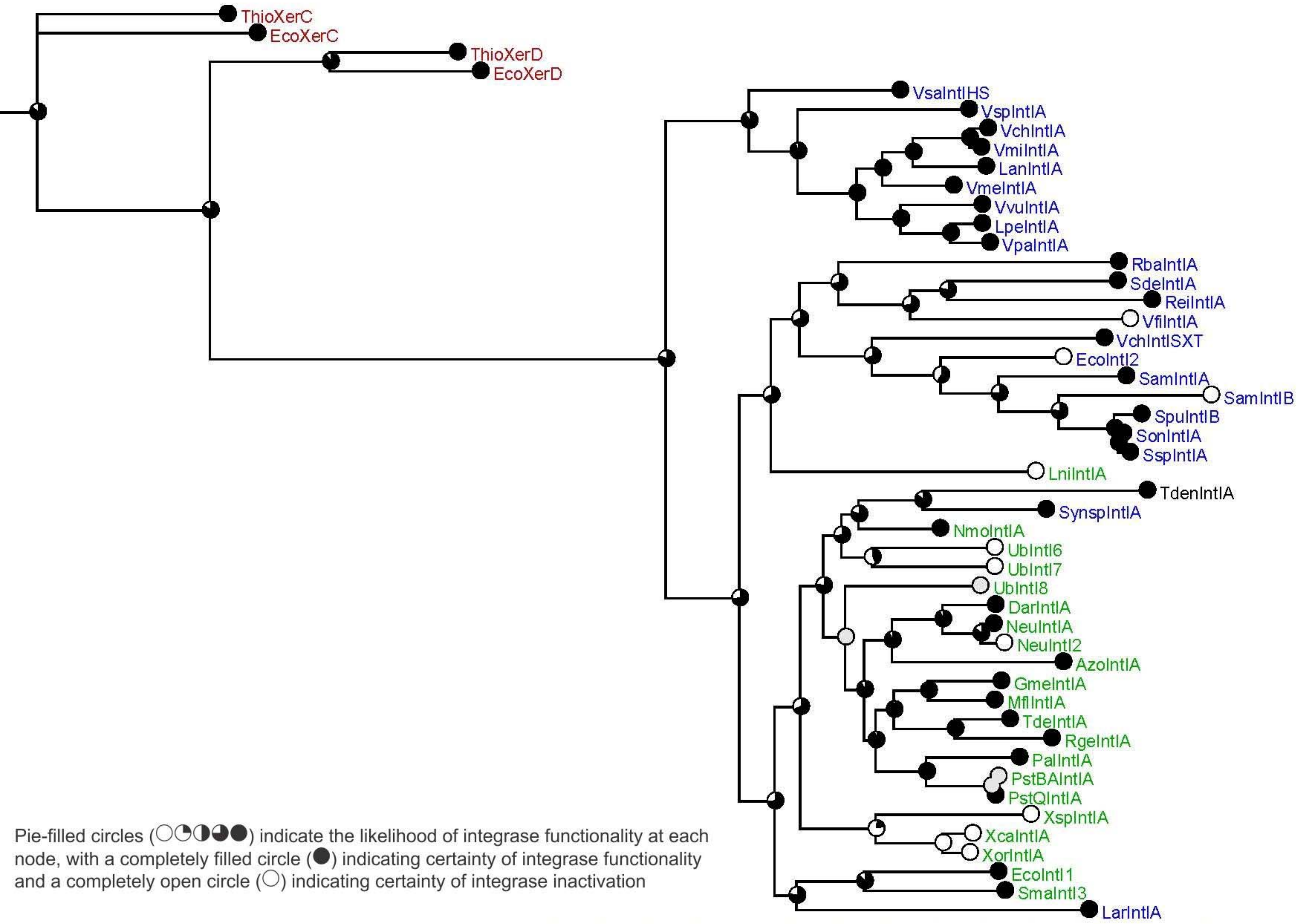

Pie-filled circles (○◔◑◕●) indicate the likelihood of integrase functionality at each node, with a completely filled circle (●) indicating certainty of integrase functionality and a completely open circle (○) indicating certainty of integrase inactivation
