# Supplementary material for: Prevalence of SOS-mediated control of integron integrase expression as an adaptive trait of chromosomal and mobile integrons
Source: Mob DNA. 2011 Apr 30;2:6. doi: 10.1186/1759-8753-2-6 (PMC3108266; doi:10.1186/1759-8753-2-6)
Supplement: Additional file 12 — Oligonucleotides used in this work. [file 1759-8753-2-6-S12.DOC]

Additional file 12: Oligonucleotides used in this work

| **name** | **Sequence (5’→3’)** | **Application** |
| --- | --- | --- |
| P1_sulA | TTAATGATACAAATTAGAGTGAATTTTTAGCCCGGAAAGTTGTCTCGTGGCGTGAGAGGAGTGTAGGCTGGAGCTGCTTC | P1 primer for the construction of UA6189 strain |
| P2_sulA | ATGTACACTTCAGGCTATGCACATCGTTCTTCGTCGTTCTCATCCGCAGCAAGTAAAATTATGGGAATTAGCCATGGTCC | P2 primer for the construction of UA6189 strain |
| P1_lexA | ATGAAAGCGTTAACGGCCAGGCAACAAGAGGTGTTTGATCTCATCCGTGATCACATCAGCGTGTAGGCTGGAGCTGCTTC | P2 primer for the construction of UA6189 strain |
| P2_lexA | TTACAGCCAGTCGCCGTTGCGAATAACCCCAACCGCCAGCCCTTCAATGGTGAAGCTCTGATGGGAATTAGCCATGGTCC | P2 primer for the construction of UA6189 strain |
| NdelexAVpa | *CATATG*AAGCCGTTAACGCCACGCC^a^ | Upper primer for cloning the *V.parahaemolyticus* *lexA* gen in pET15b overexpression vector |
| XholexAVpa | *CTCGAG*TTACATCCAATCGGTATTG^a^ | Lower primer for cloning the *V.parahaemolyticus* *lexA* gen in pET15b overexpression vector |
| NdelexAEco | *CATATGA*AAGCGTTAACGGCCAGGC^a^ | Upper primer for cloning the *E. coli* *lexA* gen in pET15b overexpression vector |
| XholexAEco | *CTCGAG*TTACAGCCAGTCGCCGTTGC^a^ | Lower primer for cloning the *E. coli* *lexA* gen in pET15b overexpression vector |
| wtintVpaF | AAAAAGCATGATAACTGGGCCAGTATTGATAAATTACAACACCTGTATAAATAAACAGACTTATAATATTATGAAAAGTCAATTTCTGCTAAGTGTAAAA | Synthetic oligo to obtain the Pint1^-^ EMSA probe |
| wtpintVpaR | ATTTTACACTTAGCAGAAATTGACTTTTCATAATATTATAAGTCTGTTTATTTATACAGGTGTTGTAATTTATCAATACTGGCCCAGTTATCATGCTTTT | Synthetic oligo to obtain the Pint1^-^ EMSA probe |
| wtpint1-pMURF | AGTAACGGCGCAGTGGCGGTTTTCATGGCTTGTTATGACTGTTTTTTTGTACAGTCTATGCCTCGGGCATCCAAGCAGCAAGCGCGTTACGCCGTGGGTC | Synthetic oligo to obtain the P_int1-_EMSA probe |
| wtpint1-pMURR | AGACCCACGGCGTAACGCGCTTGCTGCTTGGATGCCCGAGGCATAGACTGTACAAAAAAACAGTCATAACAAGCCATGAAAACCGCCACTGCGCCGTTAC | Synthetic oligo to obtain the P_int1-_ EMSA probe |
| wtpint1+pMURF | AACGGCGCAGTGGCGGTTTTCATGGCTTGTTATGACTGTTTTTTTGGGGTACAGTCTATGCCTCGGGCATCCAAGCAGCAAGCGCGTTACGCCGTGGGT | Synthetic oligo to obtain P_int1+_ EMSA probe |
| wtpint1+pMURR | AACCCACGGCGTAACGCGCTTGCTGCTTGGATGCCCGAGGCATAGACTGTACCCCAAAAAAACAGTCATAACAAGCCATGAAAACCGCCACTGCGCCGT | Synthetic oligo to obtain P_int1+_ EMSA probe |
| dxs_upVpa | AGTGCTTTCCGGTAGTCTTTA | Upper primer of *V. parahaemolyticus dxs* gene for quantitative real time RT-PCR assays |
| dxs_dwVpa | AACCTTTGCCTTTCTTAGTCA | Lower primer of *V. parahaemolyticus dxs* gene for quantitative real time RT-PCR assays |
| dxs_upEco | GACGAACTGCGCCGCTATT | Upper primer of *E. coli dxs* gene for quantitative real time RT-PCR assays |
| dxs_dwEco | CCGGCACTGATGGAGGTTGAT | Lower primer of *E. coli dxs* gene for quantitative real time RT-PCR assays |
| EcoInt_up | AAACCGAGGATGCGAACCACTT | Upper primer of pMUR050 *int* gene for quantitative real time RT-PCR assays |
| EcoInt_dw | TTACCAACCGAACAGGCTTATG | Lower primer of pMUR050 *int* gene for quantitative real time RT-PCR assays |
| VpaInt_up | CTGAATGCTATTTCGTTTTTAT | Upper primer of *V. parahaemolyticus int* gene for quantitative real time RT-PCR assays |
| VpaInt_dw | CACCTTTCCCTTGCCAGACT | Lower primer of *V. parahaemolyticus int* gene for quantitative real time RT-PCR assays |
| RecAEco_up | CCTTGCGGCACGTATGATGA | Upper primer of *E. coli recA* gene for quantitative real time RT-PCR assays |
| RecAEco_dw | CACCACGTTTTCGCCCTCTTT | Lower primer of *E. coli recA* gene for quantitative real time RT-PCR assays |
| RecAVpa_up | AAGTAGCATTTCACGCAGTTTG | Upper primer of *V. parahaemolyticus recA* gene for quantitative real time RT-PCR assays |
| RecAVpa_dw | AGAAGGCGATGAAGTTGTAGGT | Lower primer of *V. parahaemolyticus recA* gene for quantitative real time RT-PCR assays |

^a^ *Nde*I or *Xho*I endonuclease restriction sites included in the oligonucleotide sequences are shown in italics.
