# Supplementary material for: Prevalence of SOS-mediated control of integron integrase expression as an adaptive trait of chromosomal and mobile integrons
Source: Mob DNA. 2011 Apr 30;2:6. doi: 10.1186/1759-8753-2-6 (PMC3108266; doi:10.1186/1759-8753-2-6)
Supplement: Additional file 13 — Strains and plasmids used in this work. [file 1759-8753-2-6-S13.DOC]

Additional file 13. Strains and plasmids used in this work

| **name** | **relevant characteristics genotype** | **reference** |
| --- | --- | --- |
|  |  |  |
| ***Bacterial strains:*** | |  |
| ATCC17802 | *V. parahaemolyticus* ATCC17802 wild type strain | From ATCC collection |
| K12 | *E. coli* K12 wild type strain | From DSMZ collection |
| UA6189 | *E. coli* K12 Δ*sulA* Δ*lexA* | This work |
| UA10001 | *V. parahaemolyticus* ATCC17802 *ΔlexA* | This work |
|  |  |  |
| **Plasmids** |  |  |
| pMUR050 | Vector carrying two *intI* copies controled by P_int1-_ and P_int1+_ promoter region | (Gonzalez-Zorn *et al,* 2005) |
| pUA1105 | pBBR1MCS containing a 2455 bp pMUR050 fragment including *intI1* and *ant3'9* genes and the P_int1-_ promoter region | This work |
| pUA1106 | pBBR1MCS containing a 2526 bp pMUR050 fragment including the *intI2* and *ant3'9* genes and P_int1+_ promoter region | This work |
